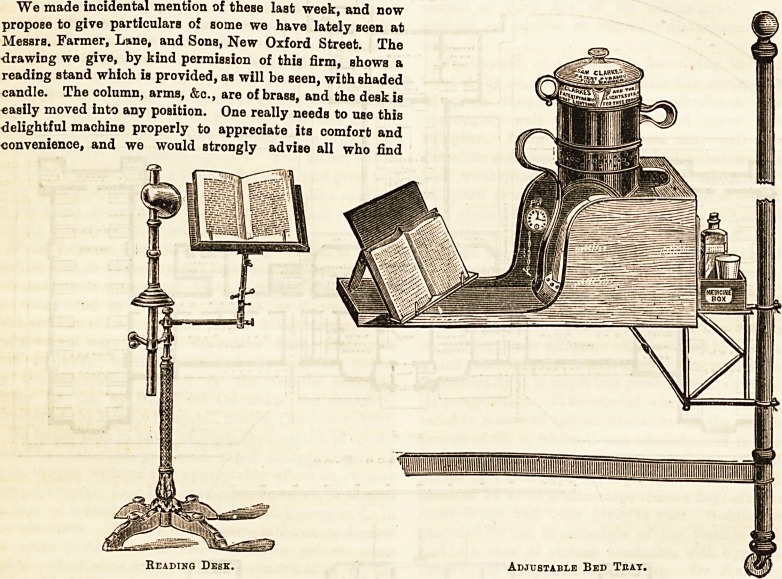# Reading Stands

**Published:** 1893-05-27

**Authors:** 


					PRACTICAL DEPARTMENTS.
READING STANDS.
We made incidental mention of these last week, and now
propose to give particulars of some we have lately seen at
Messrs. Farmer, Lane, and Sons, New Oxford Street. The
drawing we give, by kind permission of this firm, shows a
reading stand which is provided, as will be seen, with shaded
candle. The column, arms, &c., are of brass, and the desk is
easily moved into any position. One really needs to use this
delightful machine properly to appreciate its comfort and
convenience, and we would strongly advise all who find
reading at night somewhat of an effort, to invest in one of
them and see if the pleasant arrangement of light and the
comfortable position of book or paper does not make a con-
siderable difference. The desk shown in the illustration is
strongly made, and stands firm and steady, being provided with
somewhat massive feet. We notice that all the reading stands
made byMessrs.Farmer and Lane possess this letter advantage.
A very nice looking stand made in walnut or mahogany has
a email round table, and in addition to the book rest is pro-
vided with a writing desk, both revolving on a pivot, and
the stand itself being telescopic, the whole arrangement may
be heightened or lowered at pleasure. Castors, lamps, or a
small round table may be fitted to any of the stands. Under
such conditions as these an invalid will be able to read in
absolute comfort. The advantage of the light being so
arranged as to fall on the book or writing materials, while
screened from the eyes, is one which cannot be^.too strongly
imisted on, not only for invalids but for anyone in the habit
of reading or writing at night. We are sure that all who
have once made the trial will join us in strongly recommend-
ing the Btands we have described.
ADJUSTABLE BED TRAY.
The sketch we give below shows a bed-tray which will be
a great boon to sick folk. As will be seen, the tray fixes
firmly on the bedstead, and is so constructed to hold the
well-known " Pyramid " nursery lamp that its light is effec-
tually shaded from the patient and thrown on to the book.
This most convenient apparatus is a patent of Messrs. Clarke,
a familiar name in many households, where his lamps and
food-warmers have been the comfort) of nursery or sick-room.
The tray and iron bracket may be had for the small sum of
15s., the book-rest and the medicine-box costs 6s. extra.
Brass brackets and supports for book-rest, 5s. extra. Messrs.
Farmer, Lane, and Co., New Oxford Street, are agents for
this invention of Messrs. Clarke, and it can be obtained from
them, as also the latest design of the Pyramid Food Warmer.
This last is probably tco well known to need description.
We warmly recommend this " Adjustable Bed Tray '' to the
notice of nurses.
We made incidental mention of these last week, and now
propose to give particulars of some we have lately seen at
Messrs. Farmer, Lane, and Sons, New Oxford Street. The
drawing we give, by kind permission of this firm, shows a
reading stand which is provided, as will be seen, with shaded
candle. The column, arms, &c., are of brass, and the desk is
?asily moved into any position. One really needs to use this
delightful machine properly to appreciate its comfort and
convenience, and we would strongly advise all who find
Reading Desk,
Adjustable Bed Tray.

				

## Figures and Tables

**Figure f1:**